# Asxl1 loss in mice leads to microcephaly by regulating neural stem cell survival

**DOI:** 10.1080/19768354.2025.2481979

**Published:** 2025-04-23

**Authors:** Hyeju Kim, A.-Reum Kim, Sukyoung Byun, Soo-Jong Um

**Affiliations:** Department of Integrative Bioscience and Biotechnology, Sejong University, Seoul, South Korea

**Keywords:** Additional sex comb-like1 (Asxl1), Bohring-Opitz Syndrome (BOS), microcephaly, neural stem cells (NSCs), Ezh2

## Abstract

Additional sex comb-like 1 (ASXL1) is a chromatin-associated factor essential for transcriptional regulation. De novo truncating mutations in the *ASXL1* gene are linked to Bohring-Opitz syndrome, a developmental disorder characterized by microcephaly; however, the role of Asxl1 in brain development remains unclear. In this study, we demonstrate that Asxl1 deletion in mice induces microcephaly, primarily caused by a reduction in the size and number of cortical neurons. Asxl1 ablation disrupts neural stem cell (NSC) maintenance, as evidenced by decreased proliferation and increased apoptosis. Transcriptomic analysis of Asxl1-deficient NSCs revealed 4,635 differentially expressed genes, including 2,262 upregulated and 2,373 downregulated genes. Gene ontology analysis indicated that Asxl1 regulates NSC survival through the histone methyltransferase Ezh2, a core component of the Polycomb Repressive Complex 2 (PRC2). Inhibition of H3K27me3 using GSK343 significantly reduced the viability of wild-type NSCs, but had a markedly diminished effect on Asxl1-deficient NSCs. Furthermore, Ezh2 target genes associated with apoptosis, such as *Epha7* and *Osr1*, were upregulated in wild-type NSCs following GSK343 treatment but not significantly affected in Asxl1-deficient NSCs. These findings establish Asxl1 as a critical regulator of NSC survival and neurogenesis via Ezh2-mediated chromatin modification and provide insights into the mechanisms underlying microcephaly in developmental disorders.

## Introduction

Neurogenesis, the process of cell division and differentiation within the developing brain, is a tightly regulated sequence of events crucial for normal brain development. In mice, neuroepithelial progenitor cells (NEPs), the earliest form of neural stem cells (NSCs), undergo symmetric divisions around embryonic day 10 (E10), expanding the progenitor cell population (Gotz and Barde 2005). NEPs subsequently give rise to radial glial cells (RGCs), which divide asymmetrically within the ventricular zone (VZ) and subventricular zone (SVZ) to produce neurons and intermediate progenitor cells (Malatesta et al. 2000; Noctor et al. 2001, 2002, 2004; Haubensak et al. 2004; Miyata et al. 2004). Newly born neurons migrate radially from the VZ to the cortical plate (CP), traversing pre-established layers to form the laminated structure of the cortex. Early-born neurons populate the deeper cortical layers, while later-born neurons form the upper layers, with this inside-out organization being critical for cortical development (Mukhtar and Taylor 2018). Disruptions in the proliferation, self-renewal, differentiation, or migration of NSCs can result in developmental disorders, including microcephaly.

The *Additional Sex Combs-Like* (ASXL) gene family, comprising *ASXL1*, *ASXL2*, and *ASXL3*, encodes epigenetic scaffolding proteins essential for chromatin remodeling and gene regulation (Katoh 2013; Cuddapah et al. 2021; Kim et al. 2024). Among these, *ASXL1* predominantly localizes to the nucleus, where it serves as a dual regulator of nuclear receptors. Depending on the promoter context, type of nuclear receptor, or target cell type, *ASXL1* can either activate or repress transcription (Cho et al. 2006; Park et al. 2011; Park et al. 2014). Additionally, *ASXL2* has been shown to enhance transcription activated by PPARγ, LXRα, and ERα (Park et al. 2011; Park et al. 2014; Park et al. 2016), *ASXL3* functions as a repressor of LXRα-mediated transcription (Shin et al. 2014). ASXL1 also interacts with the histone methyltransferase EZH2, a core component of the Polycomb Repressive Complex 2 (PRC2), promoting H3K27 tri-methylation (H3K27me3) and thereby modulating epigenetic gene silencing (Abdel-Wahab et al. 2012; Youn et al. 2017). Although mutations in *ASXL1* are well-established in hematological malignancies, such as acute myeloid leukemia (AML), its role in developmental processes, particularly in brain development, remains poorly characterized.

Loss of *ASXL1* in mice has been associated with severe developmental phenotypes. For instance, *Asxl1* knockout mice exhibit embryonic lethality, growth retardation, kidney podocyte defects (Moon et al. 2015), impaired fibroblast proliferation (Youn et al. 2017), delayed lung maturation (Moon et al. 2018), and aberrant neural differentiation (An et al. 2019). Bohring-Opitz syndrome (BOS) is a rare developmental disorder characterized by intrauterine growth retardation, severe developmental delay, microcephaly, and distinctive facial features, including palatal anomalies (Bohring et al. 2006). De novo truncating mutations in the *ASXL1* gene have been identified as a definitive cause of BOS (Hoischen et al. 2011; Abdel-Wahab et al. 2013; Bedoukian et al. 2018; Zhao et al. 2021; Lin et al. 2023). Despite this well-established genetic association, the precise molecular mechanisms by which *ASXL1* mutations contribute to BOS pathogenesis remain poorly understood, warranting further investigation into its role in brain development and its contribution to the clinical features of BOS.

In this study, we explore the role of Asxl1 in forebrain development using *Asxl1*-deficient mice. We show that loss of Asxl1 leads to reduced cortical thickness due to decreased NSC proliferation and increased apoptosis. Transcriptomic analysis of *Asxl1*-deficient NSCs reveals significant alterations in the expression of EZH2 target genes linked to cell death, suggesting a disruption in H3K27me3-mediated gene regulation. Our findings highlight a critical role for Asxl1 in maintaining NSC survival and neurogenesis through its interaction with EZH2, providing insights into the mechanisms underlying microcephaly and developmental disorders associated with *ASXL1* mutations.

## Material and methods

### Mouse strains and breeding

*Asxl1*-deficient mice (*Asxl1 < tm1a(EUCOMM)Wtsi>*, MGI ID:2684063, EMMA ID:03996) were generated as previously described (Moon et al. 2015). All animal experiments adhered to regulations set by the Korean Council on Animal Care, with protocols reviewed and approved by the Sejong Animal Care Committee (protocol code SJ-20240111-01).

### Histological analysis

Mouse embryonic brains were harvested, fixed in 4% paraformaldehyde at 4°C overnight, washed in PBS, and cryoprotected in 30% sucrose at 4°C as described (Kim et al. 2024). Samples were embedded in OCT compound (Sakura Finetek), frozen in liquid nitrogen, and sectioned coronally (14 μm) using a cryostat microtome (Thermo Fisher Scientific). Hematoxylin and eosin (H&E) staining was performed using standard protocols.

### Immunohistochemistry and BrdU incorporation assay

For immunohistochemistry, brain sections underwent antigen retrieval in 0.01 M citric acid (pH 6.0) for 10 min, followed by blocking in 3% blocking solution (1.5% FBS, 1.5% horse serum, 0.1% Triton X-100 in PBS) for 1 h. Sections were incubated with primary antibodies overnight at 4°C. BrdU detection involved pretreatment with 2 M HCl at 37°C for 30 min. Primary antibodies included: rabbit anti-Tbr1 (1:200, abcam), rat anti-Ctip2 (1:200, abcam), mouse anti-Satb2 (1:200, abcam), rabbit anti-Ki67 (1:200, abcam), and rat anti-BrdU (1:200, abcam). Alexa Fluor secondary antibodies (Invitrogen) were used at 1:500 dilution. Slides were counterstained with Hoechst, washed, and mounted with VECTASHIELD.

For BrdU incorporation, mice received 50 mg/kg BrdU and were sacrificed after 30 min. NSCs plated on coverslips were exposed to 10 µM BrdU for 30 min, fixed in 4% paraformaldehyde, treated with 2N HCl, and processed with primary and secondary antibodies as reported (Lee et al. 2024).

### Isolation of neural stem cells and neurosphere formation assay

Neural stem cells (NSCs) were derived from the forebrain cortex of embryonic day 13.5 (E13.5) mice. Neocortices were dissected, minced, and homogenized in HBSS (GIBCO). After centrifugation (500 × g, 5 min), cells were resuspended in serum-free medium containing DMEM/F12 (1:1) (GIBCO), 2% B-27 without vitamin A (GIBCO), 1% N2 (GIBCO), 20 ng/mL EGF (GIBCO), and 20 ng/mL bFGF (R&D Systems). Cell viability was assessed by trypan blue exclusion, and cells were plated in untreated Petri dishes in a humidified incubator at 37°C with 5% CO₂. Neurospheres were dissociated using Accutase (GIBCO) and replated at a density of 2 × 10⁴ cells/60 mm dish. After 5 days, the number of dissociated cells and neurosphere diameters were measured microscopically.

### TUNEL assay

Apoptotic cells were detected using the In Situ Cell Death Detection Kit (Roche) according to the manufacturer’s protocol as previously described (Oh et al. 2024).

### Image acquisition and quantification

Fluorescence images were captured using a Leica fluorescence microscope (DFC420 C), and bright-field images with a Nikon microscope (C-DSD230). Cortical and proliferation marker quantifications were performed using FIJI (ImageJ).

### RNA isolation and real-time quantitative PCR

RNA was isolated from E13.5 passage 5 WT or *Asxl1*-/- NSCs using TRIzol (5Prime). cDNA was synthesized from 1 μg RNA with Superscript II reverse transcriptase (Invitrogen) and oligo(dT) primers (New England Biolabs). qPCR was performed using SYBR Green PCR mix (Toyobo) on a Bio-Rad CFX96 system. GAPDH served as the internal control. Primer sequences are listed in Table 1.

**Table 1.** Primers used for RT-qPCR.

Gene	Forward primer	Reverse primer
mCxcl12	GCC AAC GTC AAG CAT CTG AA	CCA GGT ACT CTT GGA TCC ACT T
mEn2	CACTGCGCCAACACTTTCTC	CCCCAAGCAGCAGATGGTTA
mEpha7	GGT AAA ATG TTT GAA GCG ACA GCA G	CCA GGC ACC AAA ACC TAC ATT G
mOsr1	GGC TCG GTG CTA AGG GAT GA	TCA CAG CCC CGG AGT TCT AC
mNkx3.2	CTGAGCGAAGAGAACGAGGG	GGCTGTGGTCGCCTGAAAC
mGabrb3	GGA GGA AGG CTT TTC GGC ATC	TGC TGG CGA TGT CGA TGT TC
mCav1	ACGTAGACTCCGAGGGACA	GTCGTTGAGATGCTTGGGGT
mFzd9	ATT GGC TAC AAC CTG ACC CG	CGT AGA GCG AGC AGA GGA AG
mSlc30a1	CGT GTT CTC TAA TGT AGC AGG TGA	CCA AGG CAT CTC CCA TCA CG
mGrid2	TGT CCA GAG GGG TCA AGT CAA	GAA TTG TGC TCT CCT GCC TCA
mGata3	GCTCCTTGCTACTCAGGTGAT	ACACGGAGGAATAAAGGGGTC
mGAPDH	GTG AAG GTC GGT GTG AAC G	TTG CCG TGA GTG GAG TCA TA

### RNA-Seq analysis

RNA quality was verified on an Agilent 2100 bioanalyzer, and quantification performed using an ND-2000 spectrophotometer. Differential expression analysis was conducted using a 1.5-fold change threshold. Gene Ontology (GO) functional classification was performed using shinyGO and SRPLOT databases.

### PI/FDA staining

To assess spheroid viability, spheroids were stained with 50 μL of 2 mg/mL propidium iodide (PI) (Sigma Aldrich) and 8 μL of 5 mg/mL fluorescein diacetate (FDA) (Sigma Aldrich) for 5 min in the dark. Staining solution was removed by PBS washes, and spheroids were imaged by fluorescence microscopy.

### MTT assay

Cell viability in response to GSK343 exposure was assessed using the MTT assay. NSCs were seeded at 1 × 10⁴ cells/100 μL per well in 96-well plates. After 24 h GSK343 treatment, 50 μL MTT solution (2 mg/mL) was added per well and incubated at 37°C for 4 h. Cell pellets were dissolved in 100 μL DMSO (Sigma), and absorbance was measured at 540 nm using a microplate reader.

### Statistical analysis

Data are presented as mean ± standard deviation from at least three independent experiments. Statistical significance was determined using paired Student's *t*-tests, with significance thresholds of *P* < 0.05 (*), 0.01 (**), or 0.001 (***).

## Results

### Asxl1 deficiency causes growth retardation and microcephaly

**Figure 1.**
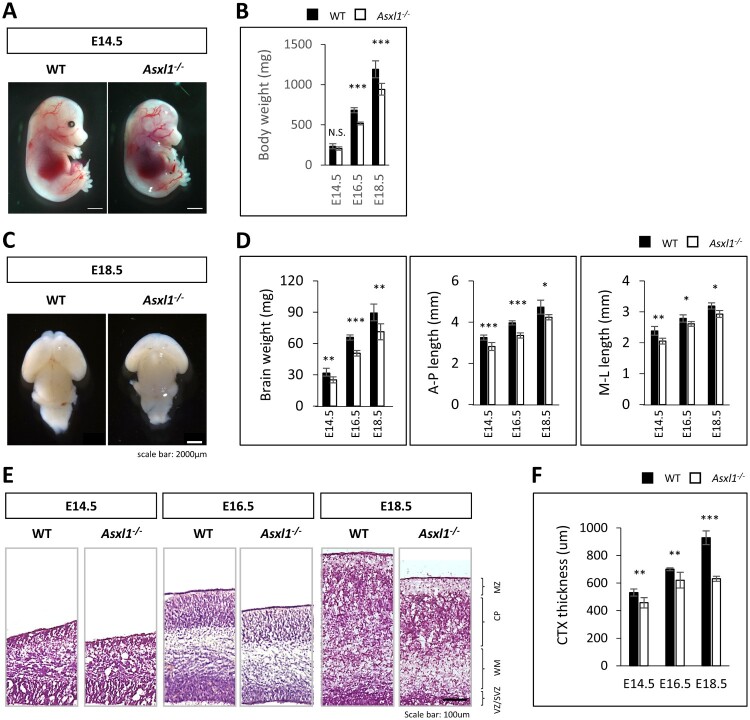
Asxl1. deficiency causes growth retardation and microcephaly. (A) Representative E14.5 embryos showing that Asxl1 deficiency leads to growth retardation and microcephaly. (B) Quantification of body weight. (C) Representative whole-mount brains from E18.5 WT and Asxl1-/- mice show reduced brain size in Asxl1-/- embryos. (D) Quantification of brain weight and cortical dimensions, including anteroposterior (A-P) and medial-lateral (M-L) lengths, comparing WT and Asxl1-/- brains (n=3). (E) Cortical thinning in Asxl1-/- mice demonstrated by methyl green-pyronin Y (MGP) staining. (D) Quantification of cortical thickness from (C) (n = 3 mice, 3 sections per mouse). Statistical significance is indicated as *p < 0.05, **p < 0.01, ***p < 0.001, or N.S. = not significant.

To examine the physiological role of Asxl1, we generated Asxl1 null (Asxl1-/-) mice as previously described (Moon et al. 2015). Asxl1-/- mice exhibited notable growth retardation, eye malformations, and small head size compared to wild-type (WT) littermates (Figure 1A). Growth retardation was evident as early as embryonic day 16.5 (E16.5; Figure 1B). In addition, Asxl1 deficiency severely disrupted normal brain development, resulting in forebrain abnormalities and olfactory bulb agenesis (Figure 1C). Analysis of brain morphology revealed an overall reduction in brain size in Asxl1-/- mice (Figure 1D). Histological examination confirmed reduced cortical thickness starting from E14.5, which persisted until E18.5 (Figure 1E, F). These findings highlight a crucial role for Asxl1 in regulating forebrain development and suggest its involvement in neurogenesis.

### Asxl1-/- mice exhibit a reduced number of cortical neurons

**Figure 2.**
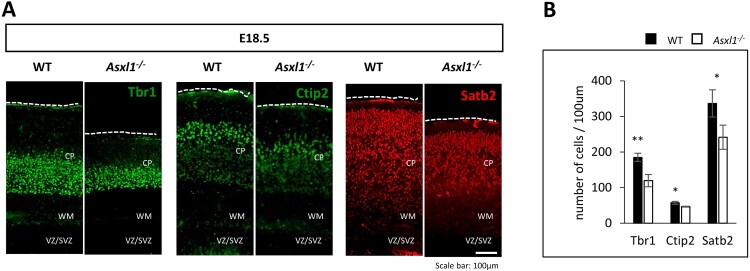
Asxl1-/- mice have a reduced number of cortical neurons. (A) Cortical sections from WT and Asxl1-/- mouse brains stained with markers for layer VI (TBR1), layer V (CTIP2) and layers IV-II (SATB2). Dashed lines indicate the pial surface, with a marked decrease in the number of neurons in Asxl1-/- brains. (B) Quantification of the number of neurons in the cortical slice per 100μm (n = 3 mice, 3 sections per mouse), with statistical significance denoted as *p < 0.05 or **p < 0.01.

To investigate whether the reduced cortical thickness in Asxl1-/- mice affects cortical neuron populations, we performed immunostaining of E18.5 forebrain coronal sections with Tbr1, Ctip2, and Satb2, which label cortical projection neurons in layers VI, V, and VI-II, respectively. A significant reduction in cortical projection neuron numbers was observed in Asxl1-/- brains compared to WT controls (Figure 2A, B). Despite this decrease, no abnormalities were detected in the distribution of neurons, suggesting that Asxl1 specifically impacts neuronal generation without affecting migration.

### Loss of Asxl1 leads to reduced proliferation of neural stem cells (NSCs)

**Figure 3.**
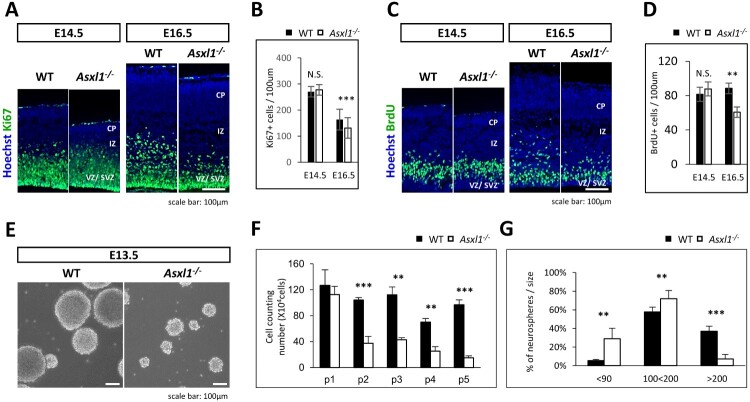
Loss of Asxl1 results in reduced proliferation of neural stem cells (NSCs) (A) Reduced proliferation was observed by immunohistochemistry (IHC) of Ki67 at E16.5. Scale bar = 100μm. (B) Quantification of (A) (n = 3 mice, 3 sections per mouse). (C) BrdU incorporation assay performed at E14.5 or E16.5 showed that the number of proliferating NSCs decreased at E16.5 but not at E14.5 in Asxl1-/- mice. Scale bar = 100 μm. (D) Quantification of (B) (n=3 mice, 3 sections per mouse). (E) Neurospheres derived from E13.5 Asxl1-/- mice grew slower and formed smaller spheres compared to WT neurospheres at passage 5. (F) After each passage, there was a gradual decrease in the number of neural stem cells derived from Asxl1-/- mice (n = 3). (G) Size distribution of E13.5 neurospheres at passage 5. WT neurospheres predominantly accumulate in the upper region, whereas a significant proportion of Asxl1-/- neurospheres tend to accumulate in the lower region (n = 3). Statistical significance is indicated as **p < 0.01, ***p < 0.001, or N.S. = not significant.

The decreased number of cortical neurons in Asxl1-/- brains prompted an investigation into NSC proliferation. Using Ki67 staining and the BrdU incorporation assay at E14.5 and E16.5, we observed significantly reduced proliferation of NSCs in the ventricular and subventricular zones (VZ/SVZ) of Asxl1-/- cortices at E16.5 (Figure 3A–D). To further assess NSC proliferation in vitro, neurospheres were generated from E13.5 cortices. Neurospheres derived from Asxl1-/- mice were smaller in size compared to those from WT mice (Figure 3E). Long-term neurosphere culture revealed a gradual reduction in the number of neurosphere-forming NSCs from Asxl1-/- mice, with the most pronounced differences observed at passage 5 (Figure 3F). Size distribution analysis of neurospheres showed that the majority of Asxl1-/- neurospheres were smaller than 90 μm, whereas WT neurospheres predominantly measured between 100–200 μm or larger than 200 μm (Figure 3G). Together, these findings demonstrate that Asxl1 is essential for maintaining NSC proliferation both in vivo and in vitro.

### Asxl1 deficiency causes apoptosis of NSCs

**Figure 4.**
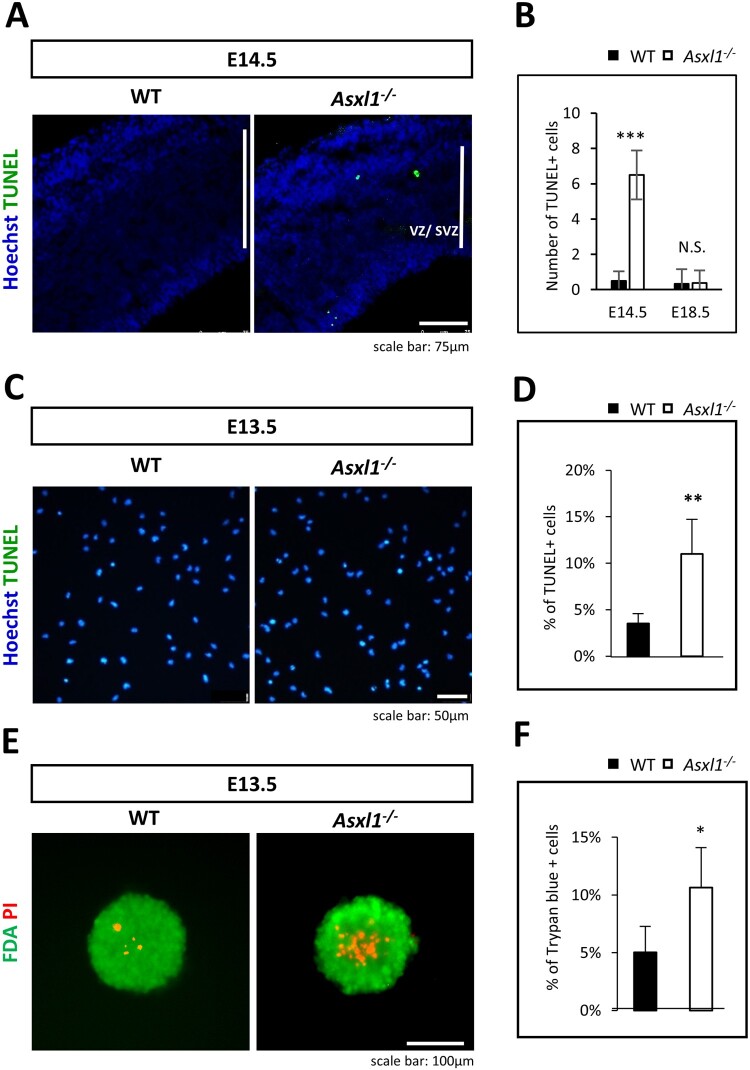
Increased number of apoptotic neural stem cells in Asxl1-/- mice. (A and B) Coronal sections of the E14.5 cortex were stained with TUNEL (green) to visualize apoptotic cells. Asxl1-/- mice showed an increased number of TUNEL-positive cells in the ventricular zone (VZ) and subventricular zone (SVZ) compared to WT mice (n = 3, 2 sections per mouse). Scale bar = 75 μm. (C) Representative image of TUNEL-positive neural stem cells isolated from E13.5 mouse. Scale bar = 50 μm. (D) TUNEL-positive cells were counted for quantification. The percentage of TUNEL positive cells was significantly increased in Asxl1-/- NSCs compared to WT (>500 cells). (E) Neurospheres were stained with FDA and PI at passage 5. Asxl1-/- neurospheres showed increased PI signals localized to the center of the spheroid. Scale bar = 100 μm. (F) The number of dead cells was determined by trypan blue staining. The analysis showed that the percentage of cell viability was increased approximately twofold compared to WT. Statistical significance is indicated as *p < 0.05, **p < 0.01, ***p < 0.001, or N.S. = not significant.

To determine whether apoptosis contributes to the reduced cortical thickness in Asxl1-/- brains, we performed TUNEL assays on embryonic brain sections. At E14.5, the number of apoptotic NSCs in Asxl1-/- brains was significantly increased compared to WT (Figure 4A, B). This difference diminished by E18.5 (Figure 4B). In vitro, TUNEL staining confirmed increased apoptosis in Asxl1-/- NSCs (Figure 4C, D). Viability assays using PI/FDA staining and trypan blue staining revealed a higher number of dead cells in Asxl1-/- neurospheres (Figure 4E, F). These results indicate that the reduction in cortical thickness observed at early developmental stages in Asxl1-/- brains is partly due to increased NSC apoptosis.

### Asxl1 regulates cell survival via the histone methyltransferase Ezh2

**Figure 5.**
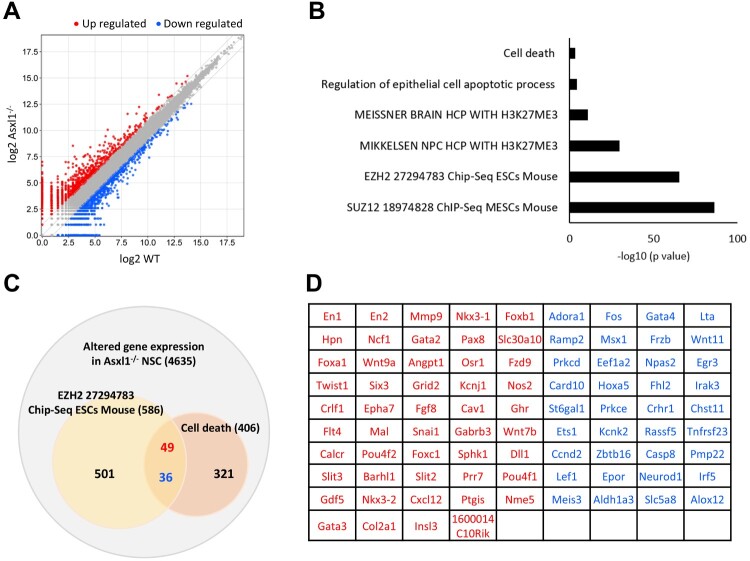
Analysis of transcriptional changes in E13.5 Asxl1-/- neural stem cells. (A) Differentially expressed genes (DEG) analysis. (B) Gene ontology (GO) analysis indicated that alterations in gene expression may affect E13.5 NSC survival through the action of histone methyltransferase EZH2. (C) Among the 4635 genes with altered expression in Asxl1-/- NSCs, 49 genes were identified as commonly up-regulated genes associated with the Gene Ontology terms ‘cell death’ and ‘EZH2’. These 85 genes are listed in (D), where genes in red are upregulated and genes in blue are downregulated in Asxl1-/- NSCs.

To investigate the molecular mechanisms underlying the role of Asxl1 in NSC survival, we conducted RNA sequencing of E13.5 NSCs. Differentially expressed gene (DEG) analysis identified 4,635 genes with at least 1.5-fold changes in expression in Asxl1-/- NSCs, with 2,262 genes upregulated and 2,373 downregulated (Figure 5A). Gene ontology (GO) analysis revealed that many of these genes are involved in NSC survival and are associated with the histone methyltransferase Ezh2, a key member of the PRC2 complex (Figure 5B). Comparative analysis identified 85 genes significantly altered in both Ezh2 ChIP-seq dataset (GSE74330) (Kloet et al. 2016) and cell death-related pathways (GO:0008219), including 49 upregulated and 36 downregulated genes (Figure 5C, D).

### Asxl1 regulates the expression of Ezh2 target genes in NSCs

**Figure 6.**
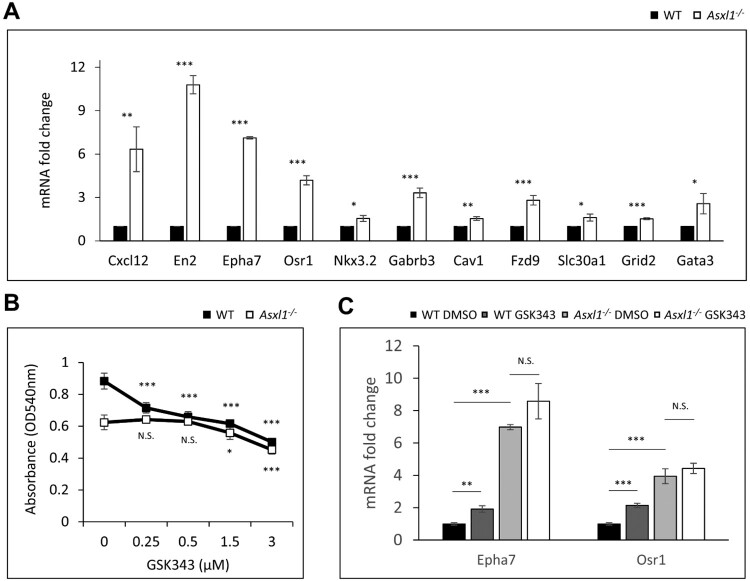
Validation of mRNA expression of genes altered by Asxl1 deletion. (A) The mRNA levels of upregulated genes in Asxl1-/- NSCs. (B) MTT analysis of cell viability after treatment with GSK343 for 24 h. (C) Epha7 and Osr1 expression levels were significantly increased in Asxl1-/- NSCs compared to WT NSCs. Epha7 and Osr1 show significantly increased expression in WT NSCs following GSK343 treatment. In Asxl1-/- NSCs, however, the expression of these genes was not significantly altered. Statistical significance is indicated as *p < 0.05, **p < 0.01, ***p < 0.001, or N.S. = not significant.

Genome-wide analysis indicated that Asxl1 modulates the expression of Ezh2 target genes to regulate NSC survival. Among the upregulated genes in Asxl1-/- NSCs, Cxcl12, En2, Epha7, Osr1, Nkx3.2, Gabrb3, Cav1, Fzd9, Slc30a1, Grid2, and Gata3 showed significant changes in expression (Figure 6A). To elucidate the functional interaction between Asxl1 and Ezh2, NSCs were treated with GSK343, a selective inhibitor of H3K27me3. WT NSCs exhibited a dose-dependent decrease in viability upon GSK343 treatment, whereas Asxl1-/- NSCs showed no significant change upto 0.5 μM of GSK343 (Figure 6B). Furthermore, GSK343 treatment significantly altered the expression of Ezh2 target genes, such as *Epha7* and *Osr1*, in WT NSCs but not significantly in Asxl1-/- cells (Figure 6C). Among other genes identified in Figure 6A, *Gird2* and *Gata3* responded similarly to GSK343 (data not shown). These results suggest that Asxl1 regulates NSC survival through Ezh2-mediated transcriptional control; however, performing Ezh2 ChIP-seq in Asxl1-/- NSCs would be better to determine the extent of Asxl1’s role in Ezh2-mediated chromatin regulation.

## Discussion

Mutations in ASXL1 have predominantly been investigated in the context of hematologic malignancies, such as acute myeloid leukemia (AML) (Asada et al. 2019; Gao et al. 2022; Yang and Agosto-Peña 2023). However, their implications for neurodevelopment have only recently begun to emerge. Neurodevelopmental syndromes, including Bohring-Opitz syndrome (BOS), associated with mutations in ASXL1, present with diverse clinical manifestations (Hoischen et al. 2011; Bedoukian et al. 2018; Zhao et al. 2021). While these phenotypes have been extensively documented, the molecular mechanisms underlying ASXL1’s role in brain development remain poorly understood. This study aimed to elucidate the role of Asxl1 in embryonic forebrain development and neural stem cell (NSC) maintenance.

Our findings demonstrate that loss of Asxl1 results in phenotypes characteristic of BOS, including microcephaly and cortical thinning. These abnormalities stem from impaired NSC proliferation and increased apoptosis during early development. Asxl1 acts as a transcriptional regulatory cofactor involved in the epigenetic control of gene expression. Consistent with our previous work (Moon et al. 2015; Moon et al. 2018), we confirm that Asxl1 deficiency disrupts essential developmental processes. In addition, earlier studies in Asxl1-deficient embryonic stem cells revealed its role in embryoid body formation and neurogenesis by modulating gene expression (An et al. 2019). Asxl1 also cooperates with Ezh2, a key component of the polycomb repressive complex 2 (PRC2), to mediate H3K27me3 deposition and repress gene expression. This interaction is critical for NSC self-renewal and survival, as Ezh2 loss is known to induce apoptosis and impair stem cell properties (Sher et al. 2012; Zemke et al. 2015). Our RNA sequencing analysis of Asxl1-/- NSCs identified dysregulation of several Ezh2 target genes, including *Epha7* and *Osr1*, which are implicated in apoptosis and cell survival. Epha7 has been shown to regulate apoptotic cell death during early brain development (Depaepe et al. 2005; Park et al. 2013). Ezh2-mediated repression of *Epha7* through histone methylation at its promoter is critical for controlling its expression (Di et al. 2019). The increased expression of *Epha7* in Asxl1-/- NSCs suggests that loss of Asxl1 disrupts this repression, contributing to elevated apoptosis. Similarly, Osr1 has been implicated in cell cycle arrest and apoptosis in other contexts, such as colorectal adenocarcinoma (Zhang and Jiang 2020). The dysregulation of Osr1 in Asxl1-/- NSCs further highlights the importance of Asxl1 in maintaining NSC homeostasis. Notably, our experiments using the Ezh2 inhibitor GSK343 revealed that WT NSCs rely on Ezh2 for survival, as its inhibition led to decreased cell viability and altered apoptotic gene expression. In contrast, Asxl1-/- NSCs exhibited resistance to Ezh2 inhibition, underscoring the critical interplay between Asxl1 and Ezh2 in regulating NSC survival and apoptosis. These findings establish Asxl1 as a key epigenetic regulator in brain development, acting through Ezh2 to control the expression of genes essential for NSC maintenance. Disruption of this pathway leads to apoptosis and impaired NSC proliferation, contributing to the phenotypic manifestations of BOS. Future research should investigate the broader implications of Asxl1-Ezh2 interactions in other neurodevelopmental processes. Additionally, exploring how Asxl1 deficiency impacts other epigenetic mechanisms and pathways could provide a more comprehensive understanding of its role in NSC biology. These insights may also facilitate the development of targeted therapies for ASXL1-related neurodevelopmental disorders, including BOS.

In summary, our study highlights Asxl1 as a critical regulator of NSC survival and brain development, operating through epigenetic mechanisms involving Ezh2 and its downstream target genes. These findings expand our understanding of ASXL1’s role in neurodevelopmental syndromes and may inform therapeutic strategies for managing these conditions.
